# Men undergoing medical circumcision at a community health centre: Knowledge and attitudes

**DOI:** 10.4102/safp.v67i1.6010

**Published:** 2025-03-21

**Authors:** Charles K. Baloyi, John V. Ndimande, Cila D. Kabongo, Jean Louis M. Yoko, Beya Mpinda

**Affiliations:** 1 Department of Family Medicine and Primary Health Care, Faculty of Medicine, Sefako Makgatho Health Sciences University, Pretoria, South Africa

**Keywords:** male medical circumcision, knowledge and attitude, HIV prevention, community health, HIV/AIDS, circumcision acceptance

## Abstract

**Background:**

Human immunodeficiency viruses (HIV) and/or acquired immunodeficiency syndrome remains a significant global health issue, affecting millions of individuals worldwide. Medical male circumcision (MMC) demonstrated effectiveness in decreasing the spread of HIV, more specifically through heterosexual contact with HIV-positive partners. Studies have shown a correlation between male circumcision and lower HIV prevalence, especially in regions where circumcision is a cultural practice. The purpose of the study was to determine the knowledge and attitudes of men utilising the MMC services regarding circumcision.

**Methods:**

A cross-sectional study was conducted at a community health centre involving 164 male participants aged 18 years and above using a self-administered questionnaire. Descriptive and inferential statistics were used to determine means, frequencies and associations between knowledge and attitudes of men regarding male medical circumcision.

**Results:**

The median age of men seeking MMC services was 30 years, with hygiene cited as the primary reason by 88.4% of them. In addition, a majority (92%) were mindful of the advantages of circumcision in terms of lowering the risk of sexually transmitted infections and penile cancer (90%).

**Conclusion:**

Hygiene emerged as the primary reason for seeking MMC, contrasting with other studies where cultural and religious factors were more common. Despite significant knowledge levels regarding MMC’s health benefits, the overall attitudes towards the procedure were predominantly negative.

**Contribution:**

The study highlights a unique factor influencing the decision to undergo MMC in a community health setting and awareness of its health benefits.

## Introduction

Human immunodeficiency viruses (HIV) and/or acquired immunodeficiency syndrome (AIDS) remains a significant global health issue, affecting millions of individuals worldwide. Human immunodeficiency virus mainly targets the body’s defences by targeting CD4+ T cells, which are essential for maintaining a robust immune defence. The virus increases the susceptibility of individuals to infections and specific malignancies by weakening the immune system. Human immunodeficiency virus may advance to AIDS, the most severe and final stage of infection, in the absence of treatment.^1^ Estimates suggest that in 2022, 39 million individuals were living with HIV worldwide, with an estimated 26 million in the sub-Saharan Africa (SSA), which accounts for almost a third of all HIV infections.^1^

Medical male circumcision (MMC) is a procedure that involves the removal of the penile foreskin by surgical means and was introduced as a strategy to prevent HIV infections in males, particularly those acquired through vaginal intercourse with HIV-positive females.^2^ Since 2007, the World Health Organization (WHO) and Joint United Nations Programme on HIV/AIDS (UNAIDS) endorsed voluntary MMC as a key HIV prevention strategy, especially for countries in the SSA where HIV prevalence is high. This recommendation was re-evaluated in 2020, emphasising its importance as a public health intervention. By the end of 2019, the programmes rapidly scaled up in 15 priority countries in the SSA, resulting in over 27 million circumcisions performed as part of HIV prevention efforts.^2^ Human immunodeficiency virus is transmitted through unprotected heterosexual intercourse, as well as from mother to child during pregnancy, childbirth or breastfeeding. Epidemiological studies show lower HIV rates in countries where male circumcision is commonly practised.^3,4,5,6^

South Africa (SA) has a 12.8% HIV prevalence rate, equating to approximately 7.1 million people living with HIV.^7^ In 2010, MMC was implemented in SA through the National Strategic Plan for HIV, tuberculosis and sexually transmitted infections (STIs) 2017–2022. The National Strategic Plan suggests that MMC be included in a comprehensive package of reproductive and sexual health services. The strategic objective of SA is to increase the scale of MMC as part of combination HIV prevention in order to reach 80% of HIV-negative men between the ages of 15 and 49 years, leading to the reduction of HIV incidence.^7^

Provinces such as KwaZulu-Natal have higher HIV rates and circumcision is less common.^8^ Medical male circumcision has proven to be effective in reducing HIV transmission.^2,9^ Traditional circumcision is an integral practice among certain ethnic groups in SA, such as the Xhosa people in the Eastern Cape, where it serves as a rite of passage.^10^ It is less common among the Zulu people in KwaZulu-Natal and the Batswana people in the North West province.^11^

The decision to undergo the procedure is often influenced by individual beliefs, knowledge and attitudes.^12^ While there is a need for MMC to expand, it is essential to assess the knowledge and attitudes of males undergoing this minor surgical procedure. The aim of this study was to assess the knowledge and attitude of men using MMC services at a community health centre (CHC) regarding circumcision and its perceived benefits.

## Research methods and design

### Research design

A descriptive cross-sectional study was conducted at a CHC using self-administered questionnaire with closed-ended questions.

### Study setting

The study was conducted at a CHC in the North West province in SA located in a rural community that serves a large population of people in a mining community. The facility provides various health services, including MMC. The area is predominantly inhabited by people speaking Setswana language, with many residents relying on the facility for both routine and specialised medical care.

### Population and sample size

The study population consisted of males who utilised MMC services at the CHC from 01 August 2019 to 30 March 2020. The target population included all males requesting MMC. A sample size of 164 men was calculated using the OpenEpi sample size calculator, with a 95% confidence level, a 5% margin of error and a *p*-value of less than 0.05 to ensure statistical significance.

### Inclusion criteria

Male patients aged 18 years and above who were uncircumcised, requesting MMC services at the health facility and provided informed consent to participate in the study were included.

### Sampling and recruitment procedure

The study used convenient sampling to sample men attending the CHC for MMC services. This technique involved selecting men who met the inclusion criteria, specifically aged 18 years and above who provided consent to participate in the study. Men were recruited as they presented at the health facility for MMC services. It is important to notice that during data collection, funding for the MMC campaign was reduced, impacting the resources available for outreach, educational activities and the participation of men. Consequently, the number of men requesting MMC services declined, highlighting challenges in maintaining awareness and access to these essential health services during the study period.

The researcher approached men who arrived for circumcision services. The study objectives and all relevant information were conveyed to the men. This included being informed about voluntary participation in the study and the care received by the men not affected regardless of their participation in the study. This was performed to ensure the men were informed before participating. Those who consented were invited and completed the questionnaire provided. The researcher did not provide medical care to any of the patients during the data collection. The researcher’s role was limited to data collection, ensuring that there was no conflict of interest or influence on the medical services provided to the men. All medical care was delivered by the healthcare staff at the facility, independent of the study’s activities. This process continued until the desired sample size was reached.

### Data collection

The questionnaire used in this study was adapted from the study conducted by Mubekapi and Davies,^13^ and was further modified by the researchers to fit the context of this study. Permission to use this questionnaire was obtained from the researchers. A pilot study was conducted to assess the questionnaire’s content, participants’ understanding and identifying any potential issues; the researchers included six questions from the initial nine questions on the knowledge section and seven questions on the attitude section. The main findings of the pilot study were not included in the main results of this study.

A self-administered questionnaire was used during the collection of data, which was originally in the English language and was translated into Setswana language, which is the predominant local language, to ensure accessibility. To verify the accuracy of the translation, the questionnaire was translated back into the English language by an independent translator.

The study comprised of three sections: socio-demographic, knowledge and attitude towards circumcision. Section one included the socio-demographic distribution of participants: these included variables such as age, status of marriage, education level, status of work and orientation of sex. Section two covered six statement questions on knowledge of circumcision. Section three had seven questioning statements about the men’s opinion towards circumcision.

### Data analysis

To record the data, Microsoft Excel sheet was used, and IBM Statistical Package for Social Sciences (SPSS) version 24, New York, United States (US) was used for the purpose of conducting the analysis of the findings. While the mean was used for reporting continuous data, frequencies and percentages for summarising categorical variables.

In the knowledge section, data coding involved converting questionnaire responses into numerical values for analysis. Six questions (Q7–Q12) assessed knowledge, with response options coded as ‘1 = True’, ‘2 = False’, and ‘3 = Not sure’. Each question had a definitive correct answer. Respondents who selected the correct answer received 1 point, while incorrect responses, including ‘Not sure’, received 0 points. For questions Q7, Q9, Q11 and Q12, the correct answer was ‘True’, earning a point. For Q8 and Q10, ‘False’ was the correct answer, also resulting in a point for correct responses.

To calculate individual knowledge scores, the researcher recoded the variables and tallied the frequency of specific values within cases as follows: for questions Q7, Q9, Q11 and Q12, the coding scheme was ‘1 = 1 (Correct)’, ‘2 = 0 (Incorrect)’, and ‘3 = 0 (Incorrect)’. For question Q8 and Q10, the coding was ‘1 = 0 (Incorrect)’, ‘2 = 1 (Correct)’, and ‘3 = 0 (Incorrect)’. Knowledge global scores were rated as (1) Good (> 75%), (2) Fair (46% – 74%) and (3) Poor (< 45%).

The scores of the men’s attitude were based on a Likert scale, where men responded to attitude-related statements by indicating their level of agreement or disagreement. The responses were scored as: (1) Strongly Agree (five points), (2) Agree (four points) (3) No Opinion (three points), (4) Disagree (two points) and (5) Strongly Disagree (one point). Each participant’s responses across all attitude questions were summed to create a total global attitude score. The total possible score ranged from 0 to 35, depending on the number of questions. Based on the total score, participants’ attitudes were classified into three categories: (1) Positive Attitude: 28–35 (≥ 80%), (2) Neutral Attitude: 15–27 (43% – 77%) and (3) Negative Attitude 7–14 (≤ 40%). This scoring system helped to quantify participants’ attitudes towards MMC and categorise them as positive, neutral or negative, based on their overall responses.

To determine the extent of the relationships, the chi-square test with a confidence interval (CI) of 95% was used. The statistical significance of the results was determined by a *p*-value that was lower than 0.05.

### Ethical considerations

Before study commencement, ethical approval was granted by Sefako Makgatho Health Sciences University (reference number: SMUREC/M/247/2017:PG). The Bojanala District Health service granted authorisation to conduct the study at the health facility, and the men provided informed consent before participation in the study. During the recruitment process, the researcher assured the men of their decision to participate in the study would not affect the quality of medical care they received. Participation was entirely voluntary and men could decline to answer any questions or withdraw from the study at any time without any impact on their access to circumcision services.

## Results

### Socio-demographic characteristics

Men in this study had a mean age of 30 years, with a standard variation of 8.2 years. The youngest man was 18 years old with the eldest being 54 years. Most men were single, with 119 (72.6%) representing more than two-thirds of the participants, while those who were married made up nearly 30%. Men with a matric educational status comprised of the largest group in the study, with 103 (62.8%) participants, followed by those with tertiary education at 27 (16.5%) and secondary education at 18 (11%). More than half of the men were employed 94 (57.3%) and 62 (37.8%) were unemployed. Most men requested MMC for hygiene reasons, with 145 (88.4%) citing this as their motivation, followed by 16 (9.8%) who sought the procedure for medical reasons. Cultural reasons were the least cited for requesting MMC, with only three men (1.8%) mentioning it. Nearly all men identified as heterosexual (*n* = 157, 95.7%), while 7 (4.3%) identified as homosexual. The socio-demographic characteristics of the men who requested MMC services are outlined in Table 1.

**TABLE 1:** Socio-demographic characteristics.

Characteristic	Frequency (*n*)	%
**Age groups (years)**		
< 20	7	4.3
20–29	71	43.8
30–39	57	35.2
40–49	22	13.6
≥ 50	5	3.1
**Marital status**		
Single	119	72.6
Married	45	27.4
**Education status**		
No formal education	6	3.7
Primary	10	6.1
Secondary	18	11
Matric	103	62.8
Tertiary	27	16.5
**Employment**		
Employed	94	57.3
Unemployed	62	37.8
Self-employed	8	4.9
**Sexual orientation**		
Heterosexual	157	95.7
Homosexual	7	4.3
**Reason for requesting MMC**		
Hygiene	145	88.4
Medical	16	9.8
Cultural	3	1.8

*Note*: Age groups (years): Mean 30 ± 8.2, Min = 18, Max = 54, Quartiles: lower = 24, mid = 30, upper = 36.

MMC, medical male circumcision; Min, minimum; Max, maximum.

### Knowledge questions about medical male circumcision

Most men agreed that MMC lowers the likelihood of contracting STIs (150, 91.5%) and reduces the risk of penile cancer (148, 90.2%). In addition, most men agreed that MMC lowers the likelihood of contracting HIV, with only 12 (7.3%) disagreeing. There were 9 men (6.1%) who were not confident that MMC reduces the risk of contracting HIV. The overall distribution of knowledge about MMC is presented in Table 2.

**TABLE 2:** Knowledge about medical male circumcision.

Knowledge questions	Responses					
	Agree		Disagree		Not sure	
	*n*	%	*n*	%	*n*	%
Circumcision reduces the chances of getting STI	150	91.5	5	3.1	9	5.5
Circumcision reduces the risk of penile cancer	148	90.2	6	3.7	10	6.1
Circumcision improves penile hygiene	143	87.2	12	7.3	9	5.5
Circumcision of a man who does not have HIV reduces his chance of getting HIV	137	83.5	11	6.7	16	9.8
Circumcision of a man with HIV does not protect his partner from getting HIV	116	70.7	34	20.7	14	8.5
There is no chance for a circumcised man to get HIV	6	3.7	136	82.9	22	13.4

STI, Sexually Transmitted Infection; HIV, Human immunodeficiency virus.

### Knowledge global score

The men’s global mean knowledge score was 85.4%, with a s.d. of 21.1%. The results indicate that a significant majority of men (147, 90.7%) achieved a good knowledge score, reflecting a strong understanding of the topic. The global knowledge scores ranged from a minimum of 16.7% to a maximum of 100.0%. The global knowledge scores are presented in Table 3.

**TABLE 3:** Participants’ global knowledge about medical male circumcision.

Knowledge score	Frequency (*n*)	%
Good	147	90.7
Average	10	6.2
Poor	5	3.1

Note: Mean 85.4 ± 21.1, Min = 16.7, Max = 100, Quartiles: lower = 83.3, Upper = 100.

Min, minimum; Max, maximum.

### Socio-demographic characteristics versus knowledge scores

Table 4 compares the socio-demographic characteristics of the men with their global knowledge scores. Most men who achieved a good global knowledge score were in the age groups 30 years and above. Notably, most men who were married achieved a good global knowledge score.

**TABLE 4:** Knowledge and participants’ demographic characteristics.

Characteristics	Knowledge						*P*
	Good		Average		Poor		
	*n*	%	*n*	%	*n*	%	
**Age groups (years)**	-	-	-	-	-	-	0.005†
Less than 20	5	71.4	1	14.3	1	14.3	-
20–29	61	85.9	6	8.5	4	5.6	-
30–39	54	94.7	3	5.3	0	0	-
40–49	22	100	0	0	0	0	-
≥ 50	5	100	0	0	0	0	-
**Education**	-	-	-	-	-	-	0.340‡
No formal education	6	100	0	0	0	0	-
Primary education	7	70	2	20	1	10	-
Secondary education	16	88.9	2	11.1	0	0	-
Matric education	92	91	5	5	4	4	-
Tertiary education	26	96.3	1	3.7	0	0	-
**Marital status**	-	-	-	-	-	-	< 0.0001§
Single	8	6.8	104	88.9	5	4.3	-
Married	43	95.6	2	4.4	0	0	-
**Employment**	-	-	-	-	-	-	0.047¶
Employed	87	93.5	5	5.4	1	1.1	-
Unemployed	52	85.2	5	8.2	4	6.6	-
Self-employed	8	100	0	0	0	0	-
**Sexual orientation**	-	-	-	-	-	-	0.418††
Heterosexual	140	90.3	10	6.5	5	3.2	-
Homosexual	7	100	0	0	0	0	-
**Reasons for requesting MMC**	-	-	-	-	-	-	0.521‡‡
Cultural	3	100	0	0	0	0	-
Medical	15	93.7	1	6.3	0	0	-
Hygiene	129	90.2	9	6.3	5	3.5	-

Note: Good knowledge score comparison:

† , < 30 years versus ≥ 30 years;

‡ , no formal, primary secondary versus matric and tertiary;

§ , single versus married;

¶ , employed versus unemployed;

†† , heterosexual versus homosexual;

‡‡ , medical versus hygiene.

MMC, medical male circumcision.

### Attitudes towards medial male circumcision

Most men (76, 46.3%) agreed that circumcised men enjoy sex more than their uncircumcised counterparts. Approximately, 41.0% of the men were indifferent on this topic, while only 12.0% disagreed. In addition, about 35.0% of the men believed that circumcised men experience more sexual feelings than uncircumcised men, with the majority (47.0%) remaining indifferent to this assertion. Only 18.0% of the men disagreed with this viewpoint.Questions on attitudes about MMC are presented in Table 5.

**TABLE 5:** Attitudes about medical male circumcision.

Attitude questions	Responses					
	Agree		Disagree		No opinion	
	*n*	%	*n*	%	*n*	%
Circumcised men enjoy sex more than uncircumcised men.	76	46.3	20	12.2	68	41.5
Circumcised men have more sexual feelings than uncircumcised men.	58	35.4	77	47	29	17.7
Circumcision enhances reproductive ability.	60	36.6	83	50.6	21	12.8
Circumcised men can safely have sex without using a condom and don’t get infected with HIV as compared to uncircumcised men.	23	14	17	10.4	124	75.6
It is important for all males irrespective of their age to be circumcised.	99	60.4	34	20.7	31	18.9
Circumcision prevents bullying by peers.	46	28	32	19.5	86	52.4
MMC is an old practice in our community and should be reintroduced.	52	31.7	31	18.9	81	49.4

MMC, medical male circumcision; HIV, Human immunodeficiency virus.

### Global attitude score

Table 6 indicated the mean global attitude score of the men as 45.4% with a s.d. of 14.3. The minimum global attitude score was 14.3% and the maximum score was 85.7%. The global attitude lower quartile score was 28.6% and the upper score was 57.1%.

**TABLE 6:** Participants’ global attitude about medical male circumcision.

Attitude score	Frequency (*n*)	%
Positive	23	14.5
Neutral	82	51.6
Negative	54	34

*Note*: Mean = 45.4 ± 19.3, Min = 14.3, Max = 85.7, Quartiles: lower = 28.6, Upper = 57.1.

Min, minimum; Max, maximum.

### Socio-demographic characteristics versus attitude scores

Men in all age groups displayed negative attitude, particularly among those younger than 30 years. When comparing the positive attitude scores between men aged 30 years and younger with those older than 30 years, no significant differences in attitudes were found. Most men with a matric educational status had neutral 49 (49.5%) and negative attitude scores 36 (36.4%), indicating a lack of positive sentiment towards the subject. The attitude scores of men who requested MMC for various reasons were predominantly neutral. Table 7 presents the men’s demographic characteristics and their attitude about MMC.

**TABLE 7:** Attitudes and participants’ demographic characteristics.

Characteristics	Attitude						*P*
	Positive		Neutral		Negative		
	*n*	%	*n*	%	*n*	%	
**Age groups (years)**	-	-	-	-	-	-	0.422†
< 20	1	14.3	3	42.9	3	42.9	-
20–29	11	15.3	38	52.8	23	31.9	-
30–39	8	14.8	27	50	19	35.2	-
40–49	2	9.5	12	57.1	7	33.3	-
≥ 50	10	20	2	40	2	40	-
**Education**	-	-	-	-	-	-	0.112‡
No formal education	0	0	3	50	3	50	-
Primary education	0	0	6	60	4	40	-
Secondary education	2	11.1	9	50	7	38.9	-
Matric education	14	14.1	49	49.5	36	36.4	-
Tertiary education	7	26.9	15	57.7	4	15.4	-
**Marital status**	-	-	-	-	-	-	0.869§
Single	18	15.5	58	50	40	34.4	-
Married	5	11.6	24	55.8	14	32.6	-
**Employment**	-	-	-	-	-	-	0.353¶
Employed	10	16	33	53.2	19	30.7	-
Unemployed	11	12.4	45	50.6	33	37	-
Self-employed	2	25	4	50	2	25	-
**Sexual orientation**	-	-	-	-	-	-	0.344††
Heterosexual	23	15	78	51.3	51	33.6	-
Homosexual	0	0	4	57.1	3	42.9	-
**Reasons for requesting MMC**	-	-	-	-	-	-	0.720‡‡
Cultural	0	0	2	66.7	1	33.3	-
Medical	1	6.7	11	73.3	3	20	-
Hygiene	22	15.6	69	48.9	50	35.5	-

Note: Positive attitude score comparison:

† , < 30 years versus ≥ 30 years;

‡ , no formal, primary and secondary versus matric and tertiary;

§ , single versus married;

¶ , employed versus unemployed;

†† , heterosexual versus homosexual;

‡‡ , medical versus hygiene.

MMC, medical male circumcision.

### Comparison of participants’ knowledge and attitude global scores

Scores varied across both the knowledge and attitudes of participants. When comparing participants with good knowledge scores to those with positive attitudes, the results revealed that participants’ knowledge was significantly higher than their attitudes, with a *p*-value of < 0.0001.

## Discussion

The median age of men who participated in the study was 30 years, with a majority in the age group 20–39 years. The study found that older men aged 30 years and above had significantly higher knowledge scores indicating a strong correlation between age and knowledge about MMC. This finding is consistent with similar studies, which also found that older men tend to have higher knowledge regarding MMC benefits.^14,15,16^ The high knowledge scores observed in older men in this study could be attributed to their broader life experiences and possibly increased exposure to health education initiatives. This aligns with findings from previous studies, which suggested that older age correlates with better knowledge and awareness of MMC’s benefits.^17^

Regarding the motivation for seeking MMC, the majority of the participants reported that improved penile hygiene and reduced risk of penile cancer were key considerations. This finding aligns with other studies that emphasise the significance of these benefits, particularly improved penile hygiene and reduced risk of penile cancer, significantly influences men’s decision to undergo MMC.^18,19,20,21,22^

Medical male circumcision was established as an effective intervention for reducing the transmission of both HIV and STIs. In this study, most of the participants recognised that circumcision reduces the likelihood of developing STIs and acknowledged its role in decreasing the risk of HIV infection for HIV-negative males. This aligns with existing research indicating that MMC contributes to lower rates of HIV transmission and STIs.^23,24,25,26,27,28^ Equally, a number of studies have found that the knowledge of the benefits of circumcision on HIV and STIs prevention motivated the pursuit of MMC.^18,19,20,29,30,31^ The significant awareness among men regarding MMC as a means to reduce the risk of HIV and STIs in this study can be attributed to government initiatives aimed at expanding MMC services.

These efforts have been supported by collaborations between public and private health sectors, complemented by targeted promotional campaigns. Such initiatives have significantly enhanced public awareness and access to MMC services, leading to a deeper understanding of its benefits in preventing HIV and STIs. Research indicates that effective health education and outreach programmes can play a crucial role in increasing knowledge about MMC and its protective effects against HIV and STIs.^32^

Although men in this study showed varying levels of education, no significant correlation was found between education level and knowledge about MMC. This finding contrasts with results from other research, which typically shows that higher educational status is associated with increased knowledge about health benefits. For instance, studies have shown that individuals with higher education are often better equipped to understand and engage with health education programmes, leading to improved health literacy and outcomes.^33,34,35^ The lack of a significant association in this study may indicate that cultural factors or societal beliefs about circumcision can overshadow the benefits of educational attainment.^36^

Overall, men in this study showed negative attitude towards MMC with only a minority expressing a positive attitude despite generally high knowledge scores. This discrepancy suggests that factors beyond knowledge, such as cultural significance and social norms surrounding circumcision, may significantly influence attitudes. Previous qualitative and quantitative studies support this notion, indicating that cultural beliefs can sometimes outweigh medical reasoning in shaping individuals’ perspectives on circumcision. This negative attitude has been documented in both qualitative and quantitative studies.^37,38,39,40^

## Conclusion

This study identified a significant correlation between age and knowledge about MMC, with older participants exhibiting notably higher knowledge scores. This finding diverges from numerous other studies that highlight education level and employment status as crucial factors influencing knowledge about MMC; in this case, these variables did not demonstrate a significant effect.

Furthermore, the attitudes of men in this study towards MMC were predominantly negative, which contrasts with more positive attitudes reported in other studies Interestingly, while hygiene was the primary reason cited for requesting MMC in this study, previous studies often identified religion and cultural beliefs as the leading motivations. This shift emphasises the need for further investigation into the factors influencing attitudes and perceptions of MMC in various demographic groups.

### Limitations of the study

Convenient sampling was used for practicality in this setting because of the unpredictable flow of participant in the setting. The method inherently lacks the randomness that would be ideal for generalising results to a larger population. The absence of a fully randomised process introduces potential bias, as the sample may not be representative of the wider population of men seeking MMC services in different settings. This limitation is acknowledged in the study and the findings are not intended to be generalised beyond the context of the selected CHC. This limitation was partly mitigated by the large number of men who regularly visit the facility for MMC services, providing a reasonably diverse cross-section of males in terms of age, education and employment status. The researchers caution against overgeneralising the results beyond similar CHCs in rural settings.

Furthermore, participants received questionnaires before undergoing the circumcision procedure, which may have influenced their responses. Men might have answered based on what they perceived to be correct rather than their true knowledge or practices to avoid jeopardising their care. This could explain why some participants lacked opinions on attitude-related questions, possibly out of fear of compromising the quality of care they would receive. The general negativity in the attitudes observed might also stem from this concern.
